# Supplementary material for: Field Angle Tuned Metamagnetism and Lifschitz Transitions in UPt3
Source: Sci Rep. 2019 Jun 3;9:8162. doi: 10.1038/s41598-019-44602-8 (PMC6547847; doi:10.1038/s41598-019-44602-8)
Supplement: Supplementary file 1 — Suppementary Info - Field Angle Tuned Metamagnetism and Lifshitz Transitions in UPt3 [file 41598_2019_44602_MOESM1_ESM.pdf]

Supplementary Information: Field Angle Tuned Metamagnetism and Lifshitz Transitions in UPt_3

B.S. Shivaram[1], Ludwig Holleis[1], V.W. Ulrich[1], John Singleton[2] and Marcelo Jaime[2]
[1]Department of Physics, University of Virginia, Charlottesville, VA. 22904
[2]National High Magnetic Field Laboratory, Los Alamos National Labs, Los Alamos, New Mexico

Ultrasound Velocity - subtraction of quadratic field dependence

As stated in the main text, for the curves shown in fig.2a (top left panel) of the main text, which shows the change in the sound velocity, we perform a parabolic fit to only the low field portion (B< 3 T). The obtained quadratic field dependence is subtracted from each curve to produce the results shown below, fig.S1. Post subtraction the sound velocity change falls into three distinct linear regions. The transition points between these regions as defined by the intersection of the blue lines are used to construct the B-T phase diagram shown in fig.2c.

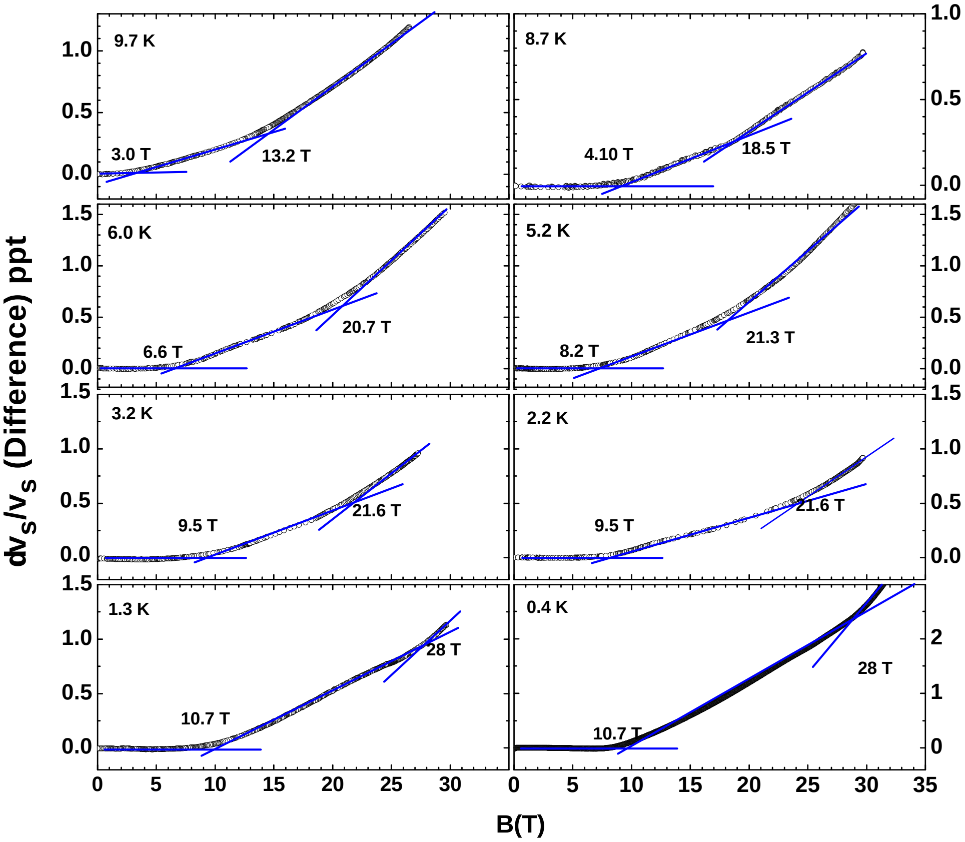

Fig.S 1: Shows the sound velocity replotted after the subtraction of an initial background B^2 dependence. Note that the sound velocity increases post both transitions.

Magneto Acoustic Quantum Oscillations - Background subtraction

In the four different panels in the figure below we illustrate step-by-step the procedure we followed to subtract the background prior to performing a Fourier transformation of the MAQO data.

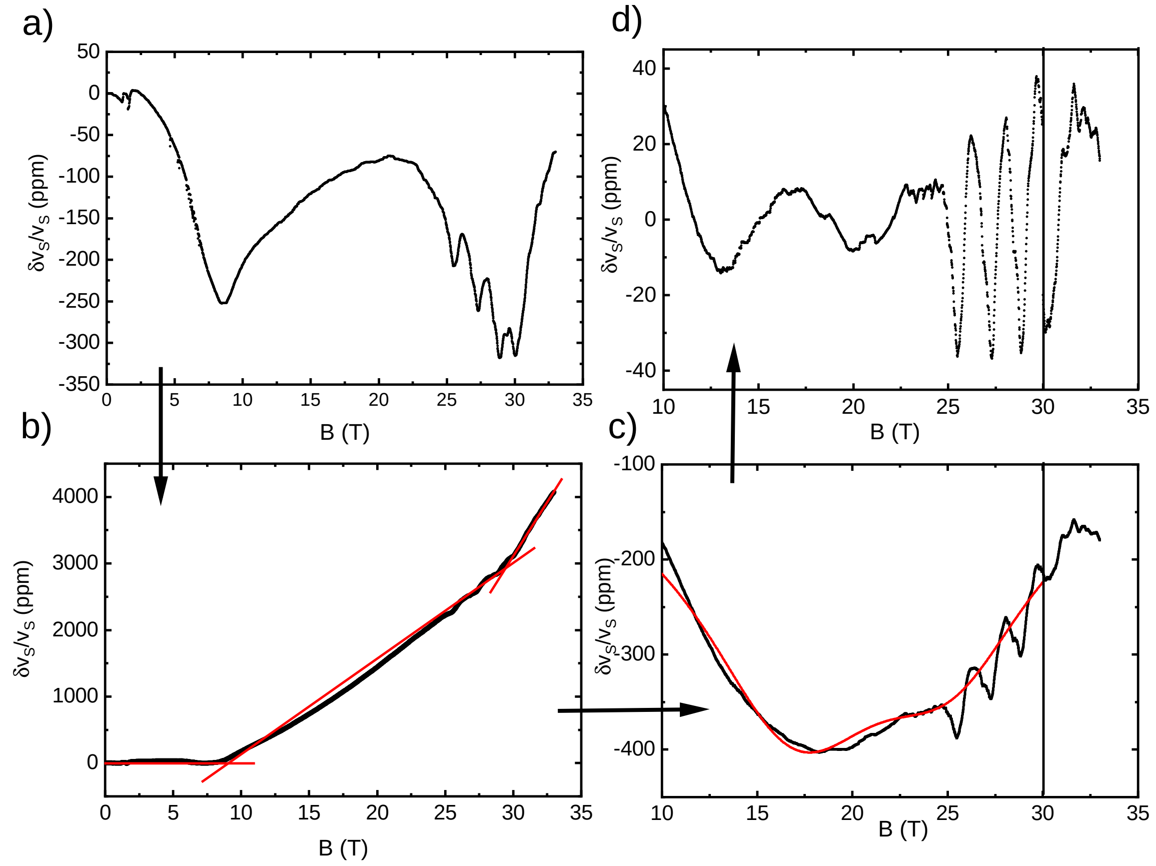

Fig.S 2: Panel shows the as obtained data on the sound velocity at T=35 mK. A subtraction of an initial background B^2 dependence results in the plot shown in panel (b). Subtraction of a linear fit to the intermediate region, 10 T to 30 T and high field region 30 T-33 T, results in a remanent part shown in panel (c). A smoothly varying double Lorentzian (inverted) is further subtracted to obtain the results shown in panel (d) on which the Fourier transformation is carried out.
